# Prevalence and determinants of undiagnosed hypertension in the Western region of Saudi Arabia

**DOI:** 10.1371/journal.pone.0280844

**Published:** 2023-03-07

**Authors:** Walaa A. Mumena, Sahar A. Hammouda, Raghad M. Aljohani, Amal M. Alzahrani, Mona J. Bamagos, Wed K. Alharbi, Bodoor M. Mulla, Hebah A. Kutbi

**Affiliations:** 1 Clinical Nutrition Department, College of Applied Medical Sciences, Taibah University, Madinah, Saudi Arabia; 2 Clinical Nutrition Department, Faculty of Applied Medical Sciences, King Abdulaziz University, Jeddah, Saudi Arabia; Christian Medical College, INDIA

## Abstract

Recent data regarding the prevalence and determinants of undiagnosed hypertension in Saudi Arabia are particularly lacking. This study aimed to investigate the prevalence of undiagnosed hypertension and to identify potential associates of hypertension risk among adults in the Western region of Saudi Arabia. Cross-sectional data for 489 Saudi adults were collected from public places in the cities of Madinah and Jeddah. Demographic, anthropometric (height, weight, waist circumference), and blood pressure (assessed by a digital sphygmomanometer) data were collected from all participants during face-to-face interviews. The American College of Cardiology and American Heart Association guidelines were used to evaluate blood pressure status. Sodium intake was assessed using a semi-validated food frequency questionnaire. The prevalence of undiagnosed, elevated blood pressure, stage I, or stage II hypertension was 9.82%, 39.5%, and 17.2%, respectively. The proportions of individuals with undiagnosed hypertension were higher among men and smokers (*p* < .001 for both). Blood pressure status was positively associated with weight, body mass index, and waist circumference among participants (*p* < .001 for all). Higher body mass index and waist circumference were associated with increased odds of stage I and stage II hypertension. Sodium intake was not associated with blood pressure status. A strikingly high prevalence of undiagnosed hypertension was observed among the study sample. National intervention programs are necessary to encourage regular screening and follow-up for the early detection and management of hypertension.

## Introduction

Hypertension (HTN) is a significant health issue, affecting more than one-third of the global population [1]. Data obtained from the National Health and Nutrition Examination Survey (2011–2012) indicated that one-third of American adults have high blood pressure [2]. In 2013, based on national data, the prevalence of prehypertension and HTN in Saudi Arabia was 40.6% and 15.2%, respectively, with 57.8% of hypertensive Saudis being undiagnosed [3].

High blood pressure is a major cause of cardiovascular disease (CVD) and stroke [4]. Untreated HTN increases the risk of life-threatening health conditions, such as stroke, heart failure, kidney disease or failure, and mortality [5]. In 2019, the World Health Organization (WHO) declared ischemic heart disease and strokes to be the leading cause of death globally and in high-income countries [6]. Similarly, In 2013, the Ministry of Health in Saudi Arabia reported that CVDs cause 42.0% of non-communicable disease-related deaths in the country [7]. To prevent the development of health consequences, health organizations recommend screening for HTN and prehypertension [8].

Available evidence suggests HTN is linked to several risk factors, including excessive sodium intake, poor fruit and vegetable intake, smoking, physical inactivity, and being overweight or obese [9, 10]. Many countries, including Saudi Arabia, are currently undertaking a nutritional transition, where westernized diets are replacing traditional diets, and individuals are becoming less active [11, 12]. This dietary shift has been associated with higher rates of obesity and comorbidities [13, 14].

The Saudi Vision 2030 is a strategic framework that includes several goals that aim to improve the quality of preventive healthcare services and healthcare system in Saudi Arabia. To achieve these goals, identifying target healthcare needs and resources for future interventions is critical, particularly in areas that lack recent and reliable data. This study aimed to investigate the prevalence of undiagnosed HTN and its association with sociodemographic factors, sodium intake, and anthropometric parameters among adults residing in the Western region of Saudi Arabia.

## Materials and methods

This cross-sectional study was conducted between January and March 2020. In order to achieve a 95% confidence level and a standardized confidence interval width of 0.20, calculations demonstrated that the minimum number of participants required was 385 [15]. Four hundred and eighty-nine adults aged between 20–50 years old were recruited from two major cities located in the Western Region of Saudi Arabia: Madinah and Jeddah. Data were collected from several public locations, such as walking paths, malls, parks, and the Jeddah Corniche. Prior to data collection, each participant signed a written consent form after aim and methods used to collect data of the study were described. The exclusion criteria included individuals previously diagnosed with HTN, diabetes, or CVD, who had been prescribed medication to treat high blood pressure, had undergone bariatric surgery, reported following a special diet, or were pregnant. Furthermore, to prevent the overestimation of HTN, individuals engaged in physical activity at the time of data collection were also excluded. This study received ethical approval from the ethical committee of the College of Applied Medical Sciences at Taibah University [SREC/AMS 2019/33/CND].

Data were collected by trained health professionals (nurses and dietitians) during face-to-face interviews. Chairs and tables were provided to participants so they could be comfortably seated during the data collection. Sociodemographic data, including sex, age, smoking status, household income, and education level, were collected from all participants. Participants were grouped into three age categories: 20–29 years old, 30–39 years old, and ≥ 40 years old.

### Clinical evaluation of blood pressure

Each participant’s blood pressure was assessed using the Cardio Simple digital sphygmomanometer (Cardio Simple, Pic Solution, Italy). All devices were calibrated and validated prior to data collection. Measurements were obtained using standardized procedures according to the instructions provided in the device manual [16]. Prior to measurements being taken, participants were asked to sit still and relax for five minutes. Blood pressure was measured three times at five-minute intervals, with the average reading calculated and documented. If a measurement appeared inappropriate, it was discarded, and a new measurement was obtained. Blood pressure cutoffs were determined based on the 2017 American College of Cardiology (ACC)/American Heart Association (AHA) guidelines [9]. Blood pressure was considered normal if the reading was < 120/80 mm Hg and elevated if systolic blood pressure (SBP) was 120–129 mm Hg and diastolic blood pressure (DBP) was < 80 mm Hg. Participants were determined to have stage I HTN if SBP was between 130–139 mm Hg or if DBP was between 80–89 mm Hg. Stage II HTN was defined as an SBP of at least 140 mm Hg or a DBP of at least 90 mm Hg.

### Assessment of sodium intake

Sodium intake was assessed using a semi-validated food frequency questionnaire (FFQ) that contained 131 items on 11 food groups. The FFQ has previously been used to assess sodium intake among adults in Saudi Arabia [17]. Participants reported their consumption frequency by selecting one of several options: 1, 2–3, 4–5, or ≥ 6 times per day; 1, 2–4, or 5–6 times per week; and less than 1 or 1–3 times per month. Participants were grouped based on their sodium intake, using the AHA recommendation of 2300 mg/day as the cutoff value: < 2300 mg/day or ≥ 2300 mg/day [18].

### Assessment of anthropometric parameters

Anthropometric measurements, including height, weight, and waist circumference (WC), were collected using standardized procedures. All measurements were assessed three times to calculate an average. Weight was measured using an electronic scale (Omron, BF508, Japan), which was placed on a hard surface. Weight was rounded to the nearest 0.1 kg. A measuring tape was placed on a straight wall to measure height, with measurements rounded to the nearest 0.5 cm. The height and weight of each participant were used to calculate their body mass index (BMI). Weight status was determined based on the WHO cutoffs: <18.5 kg/m^2^ indicated being underweight, 18.5–24.9 kg/m^2^ indicated a healthy weight; 25–29.9 kg/m^2^ indicated being overweight, and ≥ 30.0 kg/m^2^ indicated obesity [19]. The WC of each participant was measured using a fixed measuring tape and was rounded to the nearest 0.5 cm and the WHO cutoffs (male < 102 cm; female < 88 cm) as values above these cutoffs indicate a higher risk of metabolic complications [20]. At the end of the interview, each participant was provided with a card containing their anthropometric measurements and average blood pressure reading for their records.

### Statistical analysis

Descriptive data are presented as the mean (standard deviation [SD]) for continuous variables and as the frequency (percentage) for categorical variables. Associations between two categorical variables were evaluated using Fisher’s exact test. The means of normally distributed continuous variables across the blood pressure status groups (normal blood pressure = 0; elevated blood pressure = 1; stage I HTN = 2; and stage II HTN = 3) were compared using an analysis of variance (ANOVA). Multinomial logistic regression was used to estimate the unadjusted odds ratio (OR) and 95% confidence intervals (CI) for the association between blood pressure status and variables of sex (male = 1; female = 2), BMI (underweight = 1; healthy weight = 2; overweight = 3; obese = 4), and WC (within recommendation = 1; above recommendation = 2). The anthropometric measurements of the participants were correlated (r = 0.79, *p* < .001). Therefore, separate regression analyses were performed to investigate the associations of BMI (model 1) and WC (model 2) with blood pressure status. Further analyses were performed to estimate the adjusted OR (aOR), controlling for age and smoking status. All tests used were two-tailed, with the significance level set to .05. Statistical analysis was conducted using the Statistical Package for the Social Sciences (SPSS 20, SPSS Inc., Chicago, IL).

## Results

### Sample characteristics and associations with blood pressure status

Following the exclusion of 0.81% of the sample (n = 4) due to missing data, data from 489 participants were included in the final analyses. Most participants were < 30 years old, with only 17.4% (n = 85) being ≥ 40 years old. Females made up 59% of all participants (n = 288), and 58.1% (n = 284) of participants reported being married. Only 25.2% of participants were smokers (n = 123), with most smokers being male (82.3%, n = 102). Fifty-one per cent of participants (n = 247) held a university degree or higher, and 53% of participants (n = 259) earned an income of less than SAR 5,000 (< USD 1,333). The prevalence of elevated blood pressure, undiagnosed stage I HTN, and undiagnosed stage II HTN was 9.82%, 39.5%, and 17.2%, respectively. The sociodemographic characteristics of the participants, stratified by blood pressure status, are provided in Table 1. The prevalence of HTN (stage I and II combined) among males was significantly higher than among females (62.2%, n = 125 versus 52.2%, n = 152, *p* < .05). A higher prevalence of elevated blood pressure and HTN was observed among males and participants who smoked compared with females and non-smokers, respectively, (*p* < .001). A large proportion of participants (90.6%, n = 443) exceeded the AHA recommendation for daily sodium intake. However, sodium intake did not significantly differ among the different blood pressure groups.

**Table 1:** Sociodemographic and health-related characteristics of participants stratified by blood pressure status (n = 489).

	Normal blood pressure (n = 164)	Elevated blood pressure (n = 48)	Stage I hypertension (n = 193)	Stage II hypertension (n = 84)	Total (n = 489)	*p*
**Age, years, n (%)**						
20–29	111 (35.8)	35 (11.3)	118 (38.1)	46 (14.8)	310 (63.4)	.261
30–39	25 (26.6)	8 (8.51)	39 (41.5)	22 (23.4)	94 (19.2)	
≥ 40	28 (32.9)	5 (5.88)	36 (42.4)	16 (18.8)	85 (17.4)	
**Sex, n (%)**						
Male	41 (20.4)	35 (17.4)	80 (39.8)	45 (22.4)	201 (41.1)	< .001 ^a^
Female	123 (42.7)	13 (4.51)	113 (39.2)	39 (13.5)	288 (58.9)	
**Marital status, n (%)**						
Single	74 (37.0)	14 (7.00)	78 (39.0)	34 (17.0)	200 (40.9)	.272
Married	90 (31.7)	34 (12.0)	111 (39.1)	49 (17.3)	284 (58.1)	
**Education, n (%)**						
≤ High-school /Diploma	83 (34.3)	25 (10.3)	96 (39.7)	38 (15.7)	242 (49.5)	.844
University/ Postgraduate	81 (32.8)	23 (9.30)	97 (39.3)	46 (18.6)	247 (50.5)	
**Income, SAR** ^b^ **, n (%)**						
< 5000	90 (34.7)	27 (10.4)	107 (41.3)	35 (13.5)	259 (53.0)	.341
5000–10000	34 (32.4)	12 (11.4)	36 (34.3)	23 (21.9)	105 (21.5)	
> 10000	40 (32.0)	9 (7.20)	50 (40.0)	26 (20.8)	125 (25.6)	
**Smoking status, n (%)**						
Smokers	28 (22.8)	23 (18.7)	47 (38.2)	25 (20.3)	123 (25.2)	< .001 ^a^
Non-smokers	136 (37.2)	25 (6.80)	146 (40.0)	59 (15.9)	366 (74.8)	
**Sodium intake** ^c^**, n (%)**						
< 2300 mg/day	18 (39.1)	2 (4.30)	19 (41.3)	7 (15.2)	46 (9.40)	.583
≥ 2300 mg/day	146 (33.0)	46 (10.4)	174 (39.3)	77 (17.4)	443 (90.6)	

^a^Alpha was set at .05 to denote significance

^b^SR: Saudi Arabian Riyal (SAR 3.75 = $1)

^c^Based on the recommendation of the AHA.

Among the sample, 8.80% (n = 43) of participants were underweight, 38.2% (n = 187) were a healthy weight, 26.4% (n = 129) were overweight, and 26.6% (n = 130) were obese. Mean BMI and WC measurements were significantly higher among participants with HTN (*p* < .05). Weight statuses also differed significantly across the blood pressure status groups (*p* < .001). Detailed descriptions of the anthropometric measurements according to blood pressure status are provided in Table 2.

**Table 2:** Anthropometric measurements of participants stratified by blood pressure status (n = 489).

	Normal blood pressure (n = 164)	Elevated blood pressure (n = 48)	Stage I hypertension (n = 193)	Stage II hypertension (n = 84)	Total (n = 489)	*p*
**BMI (kg/m** ^**2**^ **), mean (SD)**	24.2 (5.06)	24.6 (5.67)	26.3 (5.68)	28.8 (6.42)	25.9 (5.81)	< .001^a^
**WC (cm), mean (SD)**						
Males	85.4 (12.9)	85.0 (13.6)	93.6 (20.4)	99.1 (15.5)	91.7 (17.6)	< .001^a^
Females	75.1 (10.3)	71.5 (13.2)	77.3 (11.9)	82.8 (14.6)	76.8 (12.0)	.004^a^
**Weight status, n (%)**						
Underweight	20 (46.5)	5 (11.6)	12 (27.9)	6 (14.0)	43 (8.80)	< .001^a^
Healthy weight	77 (41.2)	21 (11.2)	71 (38.0)	18 (21.4)	187 (38.2)	
Overweight	44 (34.1)	12 (9.30)	54 (41.9)	19 (41.9)	129 (26.4)	
Obese	23 (17.7)	10 (7.70)	56 (43.1)	41 (31.5)	130 (26.6)	

ANOVA and Fisher’s Exact test were used to analyze data.

^a^Alpha was set at .05 to denote significance.

### Determinants of undiagnosed hypertension

Multinomial logistic regression analyses were used to assess the association between blood pressure status and sex, BMI, and WC. The unadjusted models indicated that males had higher odds of elevated blood pressure (OR: 8.08, 95% CI: 3.90–16.7, *p* < .001), stage I HTN (OR: 2.12, 95% CI: 1.35–3.35, *p* = .001), and stage II HTN (OR: 3.46, 95% CI: 1.99–6.03, *p* < .001). An increased BMI was associated with higher odds of stage I HTN (OR: 1.07, 95% CI: 1.0–1.11, *p* = .001) and stage II HTN (OR: 1.15, 95% CI: 1.10–1.21, *p* < .001). Higher WC also was associated with higher odds of stage I HTN (OR: 1.03, 95% CI: 1.01–1.04, *p* < .001) and stage 2 HTN (OR: 1.06, 95% CI: 1.04–1.08, *p* < .001).

Multiple regression models, adjusted for participant age and smoking status, were further evaluated. In model 1, males had significantly higher odds of elevated blood pressure (aOR: 6.29, 95% CI: 2.75–14.3), stage I HTN (aOR: 1.82, 95% CI: 1.08–3.08), and stage II HTN (aOR: 2.39, 95% CI: 1.24–4.62) compared to females. A higher BMI was associated with higher odds of stage I HTN (aOR: 1.07, 95% CI: 1.02–1.12) and stage II HTN (aOR: 1.15, 95% CI: 1.09–1.21). In model 2, males had significantly higher odds of elevated blood pressure (aOR: 6.08, 95% CI: 2.54–14.5) than females, while higher WC was associated with higher odds of stage I HTN (aOR: 1.03, 95% CI: 1.01–1.05) and stage II HTN (aOR: 1.06, 95% CI: 1.03–1.08). These findings are further detailed in Table 3.

**Table 3:** Multinomial regression analyses of the associations of sex and anthropometric measurements with blood pressure status (n = 489).

BP status (Outcome)	Variable	Model 1		Model 2	
		aOR [95% CI]	*p*	aOR [95% CI]	*p*
Elevated	Male	6.29 [2.75 to 14.3]	< .001 *	6.08 [2.54 to 14.5]	< .001 ^a^
	BMI or WC	1.00 [0.93 to 1.07]	.982	0.99 [0.97 to 1.02]	.663
Stage I	Male	1.82 [1.08 to 3.08]	.025 *	1.46 [0.82 to 2.60]	.205
	BMI or WC	1.07 [1.02 to 1.12]	.005 *	1.03 [1.01 to 1.05]	.006 ^a^
Stage II	Male	2.39 [1.24 to 4.62]	.009 *	1.36 [0.64 to 2.88]	.428
	BMI or WC	1.15 [1.09 to 1.21]	< .001 *	1.06 [1.03 to 1.08]	< .001 ^a^

The reference category is normal blood pressure.

Model 1: independent variables were BMI and sex; Model 2: independent variables were WC and sex; All models were adjusted for age and smoking status.

^a^ Alpha was set at .05 to denote significance.

Associations stratified by age indicated that the younger males (20–29 years) had significantly higher odds of elevated blood pressure (aOR: 8.77, 95% CI: 3.26–23.6) and stage I HTN (aOR: 2.02, 95% CI: 1.03–3.95). A higher BMI in younger participants (20–29 years) was associated with higher odds of stage I (aOR: 1.07, 95% CI: 1.01–1.13) and stage II HTN (aOR: 1.14, 95% CI: 1.07–1.22), whereas a higher BMI among participants aged 30–39 years was only associated with higher odds of stage II HTN (aOR: 1.16, 95% CI: 1.01–1.31). Furthermore, a higher WC in younger participants (20–29 years) was associated with higher odds of elevated blood pressure (aOR: 8.54, 95% CI: 2.94–24.8), stage I HTN (aOR: 1.03, 95% CI: 1.00–1.05), and stage II HTN (aOR: 1.05, 95% CI: 1.02–1.09). Among participants aged between 30–39 years and ≥ 40 years, a higher WC was associated with higher odds of stage II HTN (aOR: 1.07, 95% CI: 1.01–1.14 and aOR: 1.06, 95% CI: 1.00–1.12, respectively). Further details are provided in Table 4.

**Table 4:** Multinomial regression analyses of the associations of sex and anthropometric measurements with blood pressure status satisfied by age group (n = 489).

BP status (Outcome)	Variable	20–29 years				30–39 years				≥ 40 years			
		Model 1		Model 2		Model 1		Model 2		Model 1		Model 2	
		aOR [95% CI]	*p*	aOR [95% CI]	*p*	aOR [95% CI]	*p*	aOR [95% CI]	*p*	aOR [95% CI]	*p*	aOR [95% CI]	*p*
**Elevated**	Male	8.77 [3.26 to 23.6]	< .001 ^a^	8.54 [2.94 to 24.8]	< .001 ^a^	7.33 [0.64 to 84.0]	.109	8.77 [0.76 to 102]	.082	0.93 [0.08 to 11.5]	.958	0.65 [0.04 to 9.99]	.754
	BMI or WC	0.99 [0.91 to 1.08]	.844	0.99 [0.96 to 1.03]	.661	0.97 [0.81 to 1.16]	.739	0.97 [0.91 to 0.1.03]	.308	1.10 [0.88 to 1.38]	.395	1.04 [0.95 to 1.13]	.438
**Stage I**	Male	2.02 [1.03 to 3.95]	.040 ^a^	1.75 [0.83 to 3.68]	.141	2.06 [0.64 to 6.60]	.227	1.72 [0.48 to 6.13]	.404	0.95 [0.24 to 3.72]	.937	0.55 [0.12 to 2.53]	.442
	BMI or WC	1.07 [1.01 to 1.13]	.012 ^a^	1.03 [1.00 to 1.05]	.028 ^a^	1.04 [0.93 to 1.17]	.453	1.00 [0.96 to 1.05]	.871	1.09 [0.96 to 1.22]	.173	1.05 [1.00 to 1.10]	.063
**Stage II**	Male	1.99 [0.83 to 4.77]	.124	1.16 [0.43 to 3.19]	.768	3.01 [0.72 to 12.6]	.131	1.45 [0.27 to 7.80]	.668	2.98 [0.68 to 13.1]	.149	1.39 [0.26 to 7.32]	.701
	BMI or WC	1.14 [1.07 to 1.22]	< .001 ^a^	1.05 [1.02 to 1.09]	< .001 ^a^	1.16 [1.01 to 1.31]	.027 ^a^	1.07 [1.01 to 1.14]	.020 ^a^	1.16 [1.00 to 1.34]	.055	1.06 [1.00 to 1.12]	.044 ^a^

Model 1: independent variables were BMI and sex; Model 2: independent variables were WC and sex; All models were adjusted for smoking status.

The reference category is normal blood pressure

^a^ Alpha was set at .05 to denote significance

## Discussion

HTN is considered a major risk factor for CVD development and associated mortality [5]. A high prevalence of HTN has previously been documented among the Saudi population [21]. However, recent data on its prevalence is lacking. The results of the current study indicated a high prevalence of elevated blood pressure and HTN among Saudi adults. HTN is a silent, asymptomatic disease, and the high prevalence of undiagnosed HTN observed in the present study indicates an urgent need for immediate action to avoid the manifestation of serious long-term complications [4, 5]. The data also indicated higher odds of elevated blood pressure and HTN in males and individuals with higher BMIs and WCs, independent of age or smoking status.

The prevalence of undiagnosed HTN observed among the study sample was high. Previous epidemiological studies have reported a lower prevalence of undiagnosed HTN among apparently healthy individuals. For example, a study conducted in India examining 3629 participants reported that approximately 26% had undiagnosed HTN [22]. However, another study conducted in Sudan that examined 1099 participants reported a higher prevalence of undiagnosed HTN (38.2%) [23]. These findings highlight the importance of identifying barriers to HTN diagnosis and developing nationwide interventions to both encourage HTN screening and increase awareness of potential HTN complications among the Saudi population.

Several studies have indicated that elevated blood pressure is more prevalent among males than females, irrespective of race and ethnicity [24, 25]. Previous studies have also reported lower levels of HTN awareness among males [25, 26]. Smoking has previously been associated with HTN in a dose-dependent manner, with longer smoking duration associated with a higher risk of HTN [27]. In the present study, elevated blood pressure was observed primarily among young males, the majority of whom were smokers. Therefore, males, in particular, should undergo routine HTN assessment, evaluation, and management, beginning at a young age, with special attention paid to those who are smokers. Intervention campaigns should be conducted to increase awareness regarding the adverse effects of smoking on cardiovascular outcomes.

Current evidence suggests that economic and nutritional transitions are occurring in various developing countries. In Saudi Arabia, fast food consumption has increased over the past few decades [28], leading to an increased intake of energy, fats, and salt [29]. Excessive sodium intake, poor consumption of dietarily important foods, including fruits, vegetables, milk, and dairy products, and more sedentary lifestyles have been reported among the Saudi Arabian population [12, 30]. Although an association between sodium intake and HTN has been reported in previous studies [10, 31], it was not linked to HTN occurrence in the current study. This may have been due to the excessive sodium consumption of sodium among the participants included in the study. Furthermore, the participants in the current study were relatively young, and previous studies have indicated that prolonged periods of high sodium intake can result in HTN, meaning this association may be more apparent among older individuals [32].

The recent dietary and lifestyle changes among the Saudi Arabian population have resulted in a higher incidence of obesity and an increased prevalence of obesity-related comorbidities, including HTN [11]. In line with previous findings, the current study reported positive associations between blood pressure status and higher BMI and WC values [33]. The pathophysiological role of obesity in the development of HTN has been extensively investigated. Various mechanisms involved in the development of oxidative stress and alterations in the renin–angiotensin–aldosterone system and sympathetic nervous system have been identified as playing key roles in elevating blood pressure in overweight and obese individuals [34].

The present study provides updated data regarding the prevalence of undiagnosed HTN and the association between blood pressure status and sociodemographic characteristics, sodium intake, BMI, and WC among Saudi adults. Such data is necessary to fill current gaps in the literature and to guide future research and interventions. However, the current study is limited by its cross-sectional design, meaning cause-and-effect relationships between blood pressure status and sociodemographic variables, sodium intake, and anthropometric measurements could not be determined. Convenient sampling method was used to collect data included in this study, which may influence the generalizability of the study findings. Furthermore, the physical activity levels of participants were not considered in the analyses. However, recent evidence suggests that the majority of Saudi individuals are living sedentary lifestyles [12].

## Conclusions

High prevalence of undiagnosed HTN was observed among the Saudi adults surveyed in this study. Nationwide data on the prevalence of HTN must be established based on recent guidelines. Males and individuals with elevated BMI or WC values were observed to be at particular risk of having undiagnosed HTN. Future longitudinal studies should be conducted to identify the specific causes of undiagnosed HTN among Saudi adults. Furthermore, intervention programs should be implemented to prevent obesity and encourage regular HTN screening and follow-ups to enable early detection and management.
